# Preliminary Assessment of the Chemical Stability of Dried Extracts from *Guazuma ulmifolia* Lam. (Sterculiaceae)

**DOI:** 10.1155/2012/508945

**Published:** 2012-01-12

**Authors:** Gisely C. Lopes, Renata Longhini, Paulo Victor P. dos Santos, Adriano A. S. Araújo, Marcos Luciano Bruschi, João Carlos P. de Mello

**Affiliations:** ^1^Programa de Pós-Graduação em Ciências Farmacêuticas, Departamento de Farmácia, Universidade Estadual de Maringá, Avenida Colombo, 5790, 87020-900 Maringá, PR, Brazil; ^2^Student of Pharmacy, Universidade Estadual de Maringá, Avenida Colombo, 5790, 87020-900 Maringá, PR, Brazil; ^3^Departamento de Fisiologia, Universidade Federal de Sergipe, Avenida Marechal Rondon, s/n, Cidade Universitária, 49100-000 São Cristóvão, SE, Brazil

## Abstract

We report the results of a preliminary estimation of the stability of the dried extract from bark of *Guazuma ulmifolia* Lam. (“Mutamba”), with and without added colloidal silicon dioxide (CSD). The physical and chemical properties and the compatibility of CSD in the extract were evaluated for 21 days of storage under stress conditions of temperature (45 ± 2°C) and humidity (75 ± 5%). Thermogravimetry (TG) was supplemented using selective high-performance liquid chromatography (HPLC) for determination of stability of the characteristic constituents (chemical markers), namely, procyanidin B2 (PB2) and epicatechin (EP). The results showed that PB2 is an appropriate compound to be used as a chemical marker in the quality control of dried extracts of *G. ulmifolia*. The stress study showed that there was no significant difference between the two formulations. However, considering the TG data and the high temperatures involved, the results suggest that CSD increases the stability of the dried extract of *G. ulmifolia*.

## 1. Introduction

The reasons for the determination of stability of pharmaceuticals are based on concern for public health. The World Health Organization (WHO) defines the stability of drugs and medicines as the ability of a pharmaceutical product to maintain its chemical, physical, microbiological, and biopharmaceutical properties within specified limits throughout the duration of product usage [1].

Several studies reported on the stability of drugs and medicines [2–4]. To the best of our knowledge, the number of stability studies of plant extracts is not the same [5–7]. Measuring the chemical stability of extracts is challenging because of their chemical complexity, which may include hundreds of different compounds. Moreover, the presence of enzymes such as glycosidases, esterases, or oxidases plays an important role in the breakdown of secondary plant metabolites.

Assessment of the chemical stability of plant extracts, many of which are promising candidates for phytomedicines, plays an important role in the process of new drug development. A variety of environmental conditions, such as light, heat, humidity, and the freeze/thaw cycle, can significantly affect the chemical stability of drugs during storage and handling. Identification of stability-affecting factors facilitates the selection of packaging material and the definition of storage and handling conditions [8].

*Guazuma ulmifolia* Lam. (Sterculiaceae), popularly known as “Mutamba”, is a tropical American plant found from Mexico to southern South America. In the popular medicine of several Latin-American countries, it is used for the treatment of burns, diarrhea, inflammations, and alopecia. Polysaccharides, epicatechin (EP), and procyanidin oligomers, such as procyanidins B2 (PB2) and B5, three trimers [procyanidin C1; epicatechin-(4**β**→6)-epicatechin-(4**β**→8)-epicatechin; epicatechin-(4**β**→8)-epicatechin-(4**β**→6)-epicatechin], and one tetramer [9, 10] have been isolated and identified from its extract. The antidiabetic properties [11, 12], hypotensive and vasorelaxant activity [13, 14], antiulcer [15, 16], antibacterial activities [17, 18], and antiviral activity [19] of the bark, aerial parts, fruits, crude extract, and fractions have been attributed to the presence of proanthocyanidins.

**Figure 1:**
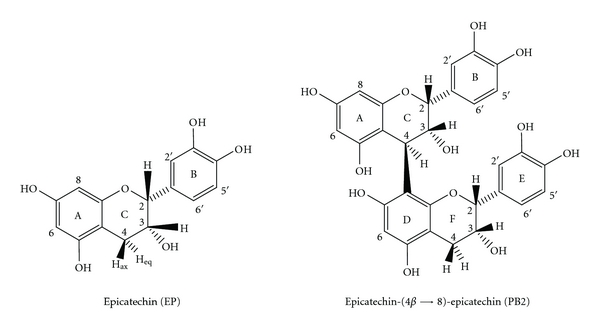
Chemical markers of the *G. ulmifolia* dried extracts.

However, there are no studies on the stability of the constituents of *G. ulmifolia* dried extracts. The determination of proanthocyanidins in bark of *G. ulmifolia* was carried out using HPLC, and it was observed that PB2 and EP compounds can be used as chemical markers for routine quality control analysis (Figure 1) [20].

The stability of the constituents in the extract of *G. ulmifolia* is important because the pharmacological properties depend on the chemical viability of the extract. As pointed out above, the procyanidins are pharmacologically active constituents of *G. ulmifolia*; however, they are unstable condensed tannins [21]. Their stability is affected by several factors such as pH, storage, temperature, chemical structure, concentration, light, oxygen, solvents, flavonoids, proteins, metallic ions, and the presence of enzymes [21]. A compatibility study of excipients is essential to develop a stable pharmaceutical dosage form, especially when the active agent is unstable.

The aim of the present study was to evaluate the chemical stability of the dried extract from the bark of *Guazuma ulmifolia* Lam. (Sterculiaceae), with and without an added pharmaceutical excipient.

## 2. Experimental

### 2.1. Plant Material

Bark of *Guazuma ulmifolia* Lam., Sterculiaceae, was collected in August 2005 in the city of Jataizinho, state of Paraná, Brazil (S 23°18′26.1′′; W 050°58′19.4′′; 377 m altitude; Garmin v.2.24). The species was identified by Professor Dr. Cássia Mônica Sakuragui. Voucher specimens are deposited in the herbarium of the Department of Biology of the State University of Maringá under number HUEM 12.051.

### 2.2. Chemicals and Reagents

All reagents and solvents were of analytical and HPLC grade, including ethyl acetate and trifluoroacetic acid (TFA) (Merck, Darmstadt, Germany). Ultra-pure water obtained by a Milli-Q UF-Plus apparatus (Millipore, Bedford, USA) with conductivity of 18.2 MΩ·cm at 25°C was used in all experiments. Epicatechin (EP) (Sigma, USA) and procyanidin B2 (PB2) (isolated and certified by spectroscopic methods at the Pharmacognosy Laboratory of Maringá State University) of the highest grade (purity > 99.0%) were used as standards. Colloidal silicon dioxide (CSD) was purchased from Degussa (Essen, Germany). All other solvents and chemicals were of analytical grade. 

### 2.3. Preparation of Extracts

Air-dried stem bark (900 g) was exhaustively extracted with 9.0 L of Me_2_CO-H_2_O (7 : 3) by turbo-extraction (Ultra-Turrax model UTC115KT; IKA; USA) for 20 min at ≤40°C. The extractive dispersion was filtered and evaporated under reduced pressure to 1.0 L and freeze-dried (Christ model Alpha 1-2, Germany), yielding 120 g of crude extract (CE). One gram of CE was dissolved in a mixture of 10 mL water and 400 mg of CSD was added [22]. This mixture (CEA) was freeze-dried under the same conditions described for CE.

### 2.4. Stability Study

CE and CEA were evaluated for thermal stability under accelerated conditions for 21 days [23]. Samples of the CE and CEA were weighed (200 mg) and packaged in opaque white polyethylene flasks with a capacity of 10 g. The CE and CEA samples were stored in a climate chamber (BINDER, model KBF 240, USA) with a constant relative humidity of 75 ± 5% and maintained at 45 ± 2°C, without direct light. Samples were analyzed at the initial time (*t*_0_) and 2, 7, 14, and 21 days after exposure to the atmospheric conditions described above.

### 2.5. HPLC Analysis

Accurately weighed 50 mg of CE and 70 mg of CEA were dissolved in 500 *μ*L water, mixed in a tube shaker, and extracted with 500 *μ*L of ethyl acetate, in a microtiter shaker at 1800 rpm (Minishaker, model MS1, IKA, USA) for 3 min (*n* = 9). Tubes were placed in a refrigerated microcentrifuge (model 5415R, Eppendorf, USA) at 4000 rpm, for 4 min at 5°C, for the total separation of the phases. The ethyl-acetate phase was separated. After evaporation of the solvent and drying under air flow, the residue was reconstituted to 10 mL with methanol : water (1 : 1) (test solution-SS). The sample was filtered through a 0.5 *μ*m membrane filter (Millipore, Bedford, USA).

The analyses were carried out using a HPLC system (Gilson, USA) consisting of a solvent delivery pump (Model 321), a variable wavelength UV/VIS detector (Model 156), a manual injection valve (Rheodyne, USA) with a 20 *μ*L loop, degasser (Model 184), and a thermostated column compartment (Model 831). Data collection and analyses were performed using UniPoint LC System software (Gilson, Villiers-le-Bel, France). A gradient was eluted on a Phenomenex Gemini C-18 column (250 mm × 4.6 mm) (Phenomenex International, USA), 5 *μ*m particle size, Phenomenex SecurityGuard (C-18 cartridge) (20 mm × 4.6 mm). The mobile phase consisted of water (0.05% TFA) as solvent A and acetonitrile (0.05% TFA) as solvent B, and both were degassed and filtered through a 0.45 *μ*m pore size filter (Millipore, Bedford, USA). Separations were affected by a gradient as follows: 0 min 13% B in A; 10 min 17% B; 16 min 18.35% B; 20 min 22.65% B; 23 min 29.81% B; 25 min 65% B; followed by a 7 min reequilibration time. The mobile-phase flow rate was 0.8 mL/min, and the injection volume was 20 *μ*L. The chromatographic runs were carried out at 28°C. UV detection was performed at 210 nm.

The purity of peaks was checked by a Diode Array Detector coupled to a Varian ProStar module (Varian, Palo Alto, CA, USA) with ProStar 210 Solvent Delivery and a ProStar 335 HPLC-DAD, comparing the UV spectra of each peak with those of authentic reference samples.

An EP reference standard stock solution of 400 *μ*g/mL was prepared in methanol : water (1 : 1). Calibration standard solutions at five levels were prepared by serially diluting the stock solution to concentrations of 10.00, 40.00, 70.00, 100.00, and 120.00 *μ*g/mL. A PB2 stock solution of 250 *μ*g/mL was prepared in methanol : water (1 : 1). Calibration standard solutions at seven levels were prepared by serially diluting the stock solution to concentrations of 20.00, 40.00, 50.00, 70.00, 90.00, 120.00, and 150.00 *μ*g/mL. The samples were filtered through a 0.5 *μ*m membrane (Millipore, Bedford, USA) prior to injection. Each analysis was repeated five times, and the calibration curves were fitted by linear regression [20].

### 2.6. Thermogravimetry (TG)

A simultaneous thermal analysis (STA) system (NETZSCH, model STA 409 PG/4/G Luxx, USA) was used for recording the TG curves of the CE and CEA. About 10 mg of sample was weighed accurately using an STA balance. The weighed sample was heated in a closed aluminum pan at a programmed rate of 10°C/min in a temperature range from 30 to 500°C under a nitrogen flow of 50 mL/min. An empty aluminum pan was used as a reference.

### 2.7. Total Tannins

The percentage of total tannins in CE and CEA at *t*_0_ and day 21 was evaluated using the Folin-Ciocalteau reagent and following a method from the British Pharmacopoeia [24]. Samples of 100 mg and 166 mg of CE and CEA, respectively, were employed. Each analysis was repeated three times.

### 2.8. Statistical Analysis

Experimental data were analyzed by one-way ANOVA, and the statistical significance of means was determined by the LSD and Tukey's HSD tests. The Dunnett test was employed to compare contents on different days of the analyses. Differences were considered significant at *P* < 0.05.

## 3. Results and Discussion

Two dried extracts of *G. ulmifolia*, prepared by different techniques (with or without CSD), were evaluated for the stability of their main components (markers): PB2 and EP.

The development of analytical conditions for the analyses herbal drugs and pharmaceutical formulations containing these extracts must necessarily go through a specific validation. In previous work, a rapid and robust LC assay for separation and quantitative analysis of PB2 and EP in extract of *G. ulmifolia* was developed. The method was validated by regulation RE 899/2003 of the National Health Surveillance Agency, Brazil, and the ICH guidelines [20].

Considering that the stability assay was developed with the aim to get an initial response about the chemical stability of the extracts for research and development purposes, the used protocol allowed the extracts to be evaluated under accelerated conditions [25].

Quantification of these markers in the samples in the test of stability was carried out using external standards (PB2 and EP). In the evaluation of linearity, based on 1/*x-*weighted linear regression analysis, the responses for both standards in related concentration ranges were linear. The calibration equations were *Y* = 818.21*x* − 2177.9 (*n* = 7, *R* = 0.9990) for PB2 and *Y* = 885.51*x* + 953.56  (*n* = 5, *R* = 0.9994) for EP. The RSDs of the slopes were ≤5% for both analytes (*n* = 5).

**Figure 2:**
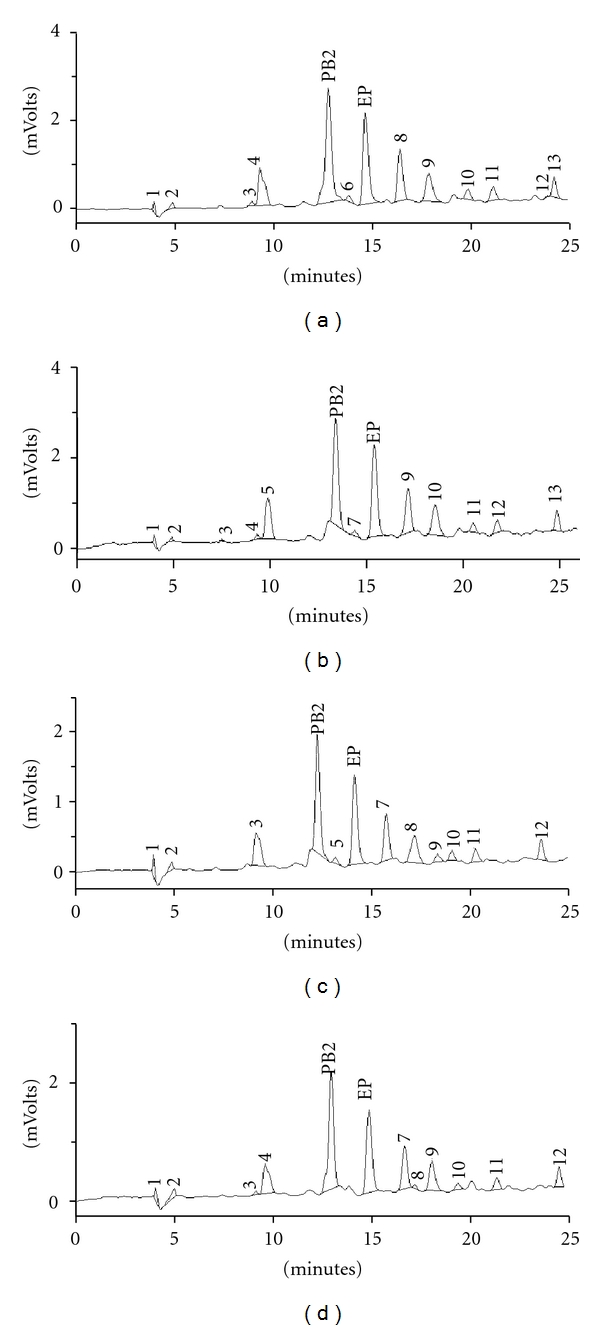
Typical HPLC chromatograms of stress test samples of the crude extract (CE) and the crude extract + colloidal silicon dioxide (CEA) of *Guazuma ulmifolia*. (a) CE at time zero; (b) CE at day 21; (c) CEA at time zero; (d) CEA at day 21.

No degradation in the CE and CEA samples under stress conditions was observed. No changes in the chromatographic profile occurred during the period of analysis (Figure 2).

The peak purity test confirmed that the PB2 and EP peaks remained homogeneous and pure throughout the stress test (data analyzed under DAD). The UV spectra of the compounds (PB2 and EP) did not change between the beginning and end of elution of their individual values, confirming the absence of degradation products.

The chemical stability assay of the CE and CEA dried-extract formulations was determined according to the concentration of PB2 and EP at a storage temperature of 45°C and 75% humidity for 21 days. The final concentration was expressed as *μ*g/mL of PB2 and EP in the dried extract (Table 1).

**Figure 3:**
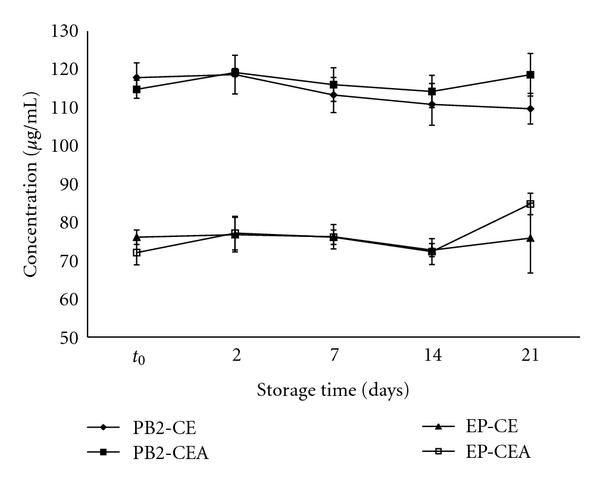
The stability of the crude extract (CE) and the crude extract + colloidal silicon dioxide (CEA) of *Guazuma ulmifolia* at 45°C, 75% relative humidity and 21 days.

Figure 3 shows the mean values of the PB2 and EP in the CE and CEA samples for each day of storage analyzed.

The PB2 content remained constant after 21 days of storage, in both the CE and CEA. The EP in the CEA showed a significant change (*P* < 0.05) in concentration from *t*_0_ to day 21. However, no significant change in the concentration of EP was observed in the CE stored under the same conditions.

**Figure 4:**
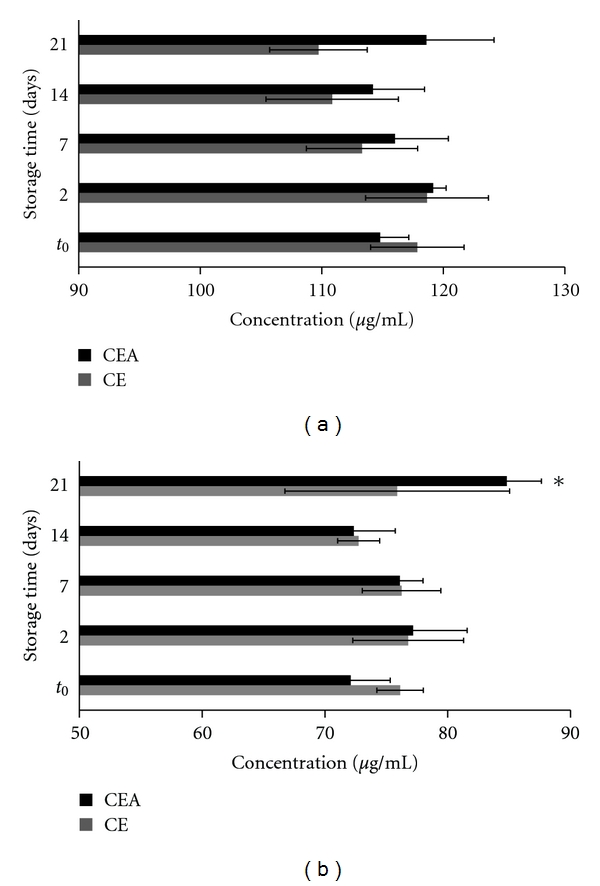
Influence of colloidal silicon dioxide on the dried-extract (CE and CEA) of *Guazuma ulmifolia.* (a) procyanidin B2 (PB2) and (b) epicatechin (EP).

Figure 4 shows the influence of the presence of the excipient in the dried extract. Apparently, the physical and chemical properties of the CSD can significantly accelerate the increase of EP in the CEA after 21 days. In relation to the concentration of PB2, there was no significant difference between the CE and CEA during the 21 days of analysis.

Proanthocyanidins are commonly composed of monomers of catechin and/or epicatechin with linkages of 4→6 and/or 4→8. Besides these, other monomers are common: gallocatechin, epigallocatechin, robinetinidin, and fisetinidin [26]. Proanthocyanidins differ structurally according to the number of hydroxyl groups present at aromatic rings and the stereochemistry of the asymmetric carbons of the heterocyclic nucleus. The presence of *O*-methylation, *O*-glycosylation, and *O*-galloylation increase the structural complexity [27].

PB2 is a dimeric proanthocyanidin with chemical linkage of the type 4**β**→8. Fletcher et al. [28] showed by NMR studies of the procyanidin peracetate that linkages 4→6 and 4→8 are found in two energetically protected conformations. Therefore, the linkage between epicatechin monomers forming the PB2 may be physically more stable.

The significant change in concentration of EP in the CEA probably occurred by physical interaction of oligomers and/or polymers of condensed tannins in the extract and CSD. This excipient has a large surface area and a high polarity of silanol groups present on its surface, which leads to adsorption of water and formation of hydrogen bonds [29], facilitated by its hygroscopic property [30]. Therefore, CSD is commonly used as a desiccant agent to protect hygroscopic chemicals and drugs from atmospheric moisture [29]. Thus, this excipient is an excellent candidate adjuvant for the stabilization of plant extracts.

Extracts rich in phenolic substances are congruent with this assumption because they are rich in hydroxyls, capable of hydrogen bond interactions. Döner et al. [31] evaluated the bonding between polyvinylpolypyrrolidone (PVP) and different classes of flavonoids. The bonding increases with the number of hydroxyl groups present in the flavonoid nucleus. Compounds that contain 7- and 4′-hydroxyl groups bond most effectively; the same principle can be extrapolated to the CEA. The increase in the concentration of EP in the CEA (Figure 4(b)) may result from an interaction by hydrogen bonding between oligomers and/or polymers of the condensed tannins and the silanol hydroxyl group of CSD.

Gore and Banker [29] observed that silica has the ability to form a monolayer adsorption of water vapor, suggesting that polar water molecules are adsorbed at specific sites on the silica surface. Oligomeric flavonols and polymers of condensed tannins may show the same pattern of connection to the CSD. Bonding of these substances with CSD would weaken the bonds within the compound, releasing monomeric substances. This would explain the statistical difference found at day 21.

However, the analyses of the total tannin content of CE and CEA at time *t*_0_ and day 21 after the stress tests showed no significant differences. The results for CE were 26.1% ± 0.5 (RSD% 2.0) and 26.6% ± 0.8 (RSD% 3.0), and for CEA were 25.0% ± 1.2 (RSD% 4.9) and 25.4% ± 0.7 (RSD% 2.8) at times *t*_0_ and day 21, respectively.

These results suggest that the physical interactions occurred in the extract CEA produced no alterations in the content of proanthocyanidin. However, they show that the quality control of extracts containing high content of phenolic compounds must be accomplished using dimeric compounds, which are more physically stable.

**Figure 5:**
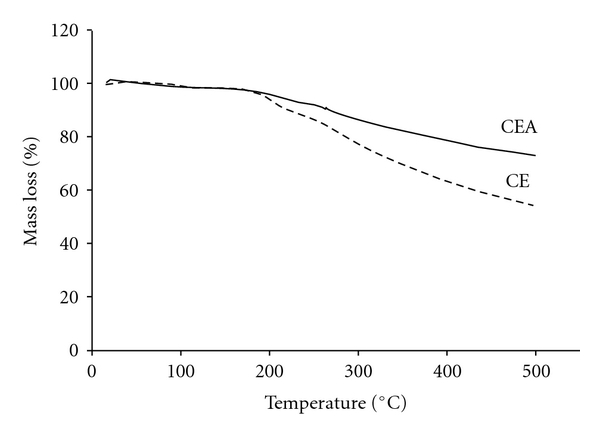
Thermogravimetry curves for the (dashed line) crude extract (CE) and (staked line) crude extract + colloidal silicon dioxide (CEA) of *Guazuma ulmifolia* at time zero.

Figure 5 and Table 2 show the TG data in the temperature range from 25 to 500°C for the CE and CEA. TG curves of CE and CEA presented a characteristic profile of elimination of water surface between 35 and 100°C, thermal stability between 100 and 185°C, following thermal decomposition. The thermal decomposition of CEA occurs in two stages, (Δ*m*_2_ = 4.80% and DTG_peak_ = 213°C and Δ*m*_3_ = 19.79% and DTG_peak_ = 340°C). A similar thermal decomposition was observed for CEA. However, TG curves of CE presented mass losses greater than those of CEA (Δ*m*_2_ = 9.70% and DTG_peak_ = 218°C and Δ*m*_3_ = 32.46% and DTG_peak_ = 336°C).

The TG analysis indicated no trend in the behavior of CE and CEA, that is, during the days of analysis, the total mass underwent no significant increase or decrease.

The analysis of these data leads us to suppose that CSD conferred some stability, because the CEA lost significantly less total mass. It can be assumed that the CEA was protected from heat to some extent by the CSD, influencing the process of degradation, with smaller percentages of loss of mass from the fusion of chemicals. Thus, we can conclude that the CSD limited the access of water to the extract and/or prevented its degradation [32, 33].

Over the 21 days of the study, there was no significant difference between the two extracts, but considering the TG data and the high temperatures involved, the data suggest that over the long term the CSD would be a good protector for the plant extracts of *G. ulmifolia*. However, further studies should be performed to confirm these results.

As a conclusion, PB2 is an appropriate compound to use as a chemical marker in quality control of the dried extract of *G. ulmifolia*. The stress test showed that the content of total tannins was unchanged. Therefore, in this 21-day screening study, proanthocyanidins in the dried extract of the *G. ulmifolia* showed good compatibility with CSD under stress conditions.

## Figures and Tables

**Table 1:** Stability of the constituents procyanidin B2 (PB2) and epicatechin (EP) of the dried extract of *Guazuma ulmifolia*. Mean ± SD (RSD %).

Days of storage	CE (mg/mg)		CEA (mg/mg)	
	PB2	EP	PB2	EP
Time zero	0.0236 ± 4.5 (3.8)	0.0152 ± 1.4 (1.9)	0.0230 ± 2.7 (2.3)	0.0144 ± 2.3 (3.2)
2	0.0237 ± 6.0 (5.1)	0.0154 ± 3.5 (4.5)	0.0238 ± 1.2 (1.0)	0.0154 ± 3.4 (4.4)
7	0.0226 ± 5.2 (4.6)	0.0152 ± 2.4 (3.2)	0.0232 ± 5.1 (4.4)	0.0152 ± 1.4 (1.9)
14	0.0221 ± 10.5 (9.4)	0.0147 ± 1.2 (1.7)	0.0228 ± 4.8 (4.2)	0.0145 ± 2.4 (3.4)
21	0.0220 ± 9.9 (9.0)	0.0152 ± 6.9 (9.1)	0.0237 ± 7.8 (6.6)	0.0170 ± 2.4 (2.8)

**Table 2:** Thermogravimetry parameters of the crude extract (CE) and the crude extract + colloidal silicon dioxide (CEA) of *Guazuma ulmifolia*.

Sample	Mass loss (%)	Days of storage				
		*t*_0_	2	7	14	21
CE	1	3.68	4.44	4.51	4.85	4.90
	2	9.70	8.44	8.98	8.38	8.27
	3	32.46	33.06	32.29	31.79	30.75

	Total mass loss (%)	45.87	46.22	46.81	45.45	44.85

CEA	1	2.31	3.54	3.11	2.07	2.36
	2	4.80	4.15	5.13	4.85	4.97
	3	19.79	19.39	20.89	18.98	19.21

	Total mass loss (%)	27.33	27.60	29.47	26.03	26.93
